# Methadone use for acute opioid withdrawal in Tshwane shelters during the COVID-19 lockdown

**DOI:** 10.4102/safp.v65i1.5708

**Published:** 2023-09-05

**Authors:** Jo-Marie A. Siemens, Urvisha Bhoora, Michelle Janse van Rensburg

**Affiliations:** 1 Department of Family Medicine, Faculty of Health Sciences, University of Pretoria, Pretoria, South Africa; 2 The Community Oriented Substance Use Programme (COSUP), Tshwane, South Africa

**Keywords:** substance use, opioid dependence, COVID-19, adherence, methadone, COSUP, homeless shelters

## Abstract

**Background:**

Temporary shelters were established for street-based people during the national level 5 coronavirus disease 2019 (COVID-19) lockdown. However, street-based substance users’ need to access substances was not addressed, resulting in large numbers of people experiencing withdrawal. The Community Oriented Substance Use Programme (COSUP) in Tshwane provided methadone to manage opioid withdrawal.

**Methods:**

A cross-sectional, descriptive study was conducted using the daily methadone dosing records from shelters in Tshwane between March 2020 and September 2020.

**Results:**

The final analysis included 495 participants, of which 64 (12.9%) were initiated on 20 mg – 30 mg of methadone, 397 (80.2%) on 40 mg – 50 mg, and 34 (6.9%) on 60 mg – 70 mg. A total of 194 (39.2%) participants continued their initiation dose for 1–2 months, after which 126 (64.9%) had their doses increased, and 68 (35.1%) had their doses decreased. Approximately 12 (2.4%) participants were weaned off methadone after 1–3 months and 46 (9.3%) after 4–6 months. In all, 100 (20.2%) participants left the shelter prematurely and did not continue with methadone. A total of 126 (25.5%) participants continued to stay in the shelters and received methadone for 6 months, with 125 (25.3%) participants leaving the shelter with continued follow-up at a COSUP site.

**Conclusion:**

This study demonstrates variability in methadone dosing regimens among shelter residents. As the lockdown measures eased, many chose to leave the shelters, while others remained to receive methadone and other services. The COSUP appears to be effective during periods of increased vulnerability, since a large number of participants were successfully followed up.

**Contribution:**

Opioid dependence is a persistent, lifelong disease. It is multifaceted with complex environmental and individual determinants. This study highlighted the use of opioid substitution therapy during a period of increased vulnerability.

## Introduction

On 11 March 2020, the World Health Organization (WHO) announced the coronavirus disease 2019 (COVID-19) outbreak as a pandemic. As part of South Africa’s response to the global pandemic, a national Level 5 lockdown was implemented at midnight on 26 March 2020. The lockdown restricted movement and enforced home isolation to prevent the spread of disease. This posed a challenge to the massive street-based population in South Africa, especially in metropolitan areas such as the City of Tshwane. In an effort to address this challenge, previously established housing facilities were activated, and temporary shelters were established to facilitate the safe removal of the homeless population off the streets of Tshwane. Restrictions brought on by the national lockdown were also challenging to substance users, as they were no longer able to access substances, increasing the burden of substance withdrawal in the shelters and facilities. In an attempt to manage withdrawal, a harm-reduction programme was implemented in these settings, using opioid substitution therapy (OST) in the form of methadone.

Opioid dependence is a persistent, lifelong disease. It is multifaceted with complex environmental and individual determinants. No simple solution exists; treatment is often longstanding and expensive and, in the majority of cases, unsuccessful.^1,2^ The United Nations Office on Drugs and Crime (UNODC) World Drug Report for 2020 showed that an estimated 269 million people used drugs at least once in 2018, of which 35.6 million were estimated to suffer from a substance use disorder (SUD). Globally, 11.6 million injecting drug users were documented, with South Africa having an estimated 140 000 injecting drug users.^3^ The most common illicit opioid used in South Africa is heroin,^4^ and South Africa is one of the top three regions in Africa most severely affected by opioid-related premature mortality.^5^ A thriving drug trade, poor border control, and reduction of opioid cost have worsened the trend of opioid use.^4^ ‘Nyaope’, also known as ‘whoonga’, ‘unga’ or ‘spices’, is the street name for a heroin-based drug that is currently ravaging urban and peri-urban communities of South Africa.^2,4,5^ Nyaope is a mixture of heroin, and other materials added as cutting agents. It is sold in powder form and can be smoked, rolled with cannabis in a ‘joint’, or liquidised for injection.^6,7,8^

The traditional management approach to opioid dependence involves the total renunciation of all opioids.^6^ Generally, the overall success of total abstinence is disappointing, even following inpatient rehabilitation. Inpatient rehabilitation, also called residential rehabilitation, describes an addiction treatment programme that is provided to patients in a residential setting. Patients reside at the residential treatment facility for the duration of their treatment programme, which may be short-term (30 days or less) or long-term (more than 30 days).^6,9^ The length of treatment time depends on the type of SUD, duration and frequency of use, and any co-occurring SUDs or mental health disorders.^9^

Inpatient rehabilitation services in South Africa are largely unattainable for the majority of users due to the high cost of treatment and the lack of the availability of the centre.^1,9^ Currently, in Gauteng province there is only one state-funded inpatient rehabilitation centre in Cullinan, with a capacity of 296 patients. The waiting list to enter this facility can be anything from 6 weeks to 5 months. Additionally, there are five non-governmental organisation (NGO)-run inpatient rehabilitation facilities throughout Gauteng, but due to the small capacity and private admission costs these tend to be inaccessible to the homeless and poor populations.^10^

Readmission to inpatient comprehensive opiate detoxification programmes is frequent.^11^ Forty-three percent of patients relapse to heroin use within 3 days after completing inpatient short-term rehabilitation, increasing to 45% after 7 days, and up to 50% after 14 days. A national treatment outcome study, performed in Johannesburg, South Africa, showed that 60% of users relapsed after 90 days.^1,2,12^ Relapse is influenced by high prevalence of psychopathology. Studies have shown that up to 80% of heroin users suffer from a combination of either mood, anxiety, or personality disorders.^2,13^ Other factors related to continued heroin use include: a longer history of heroin consumption, a shorter treatment duration, a lack of family support, and being human immunodeficiency virus (HIV)-positive.^1,2,4,5,13^

Opioid detoxification, the most widely used treatment modality, is associated with high relapse rates, and has been falling out of favour as it is shown to be ineffective.^2,4,11^ Opioid substitution therapy as maintenance, however, has shown promising results, with only 20% – 30% of users still partaking in frequent heroin use. Users on an OST programme have been able to stabilise their lifestyle, rebuild relationships, find employment (or at least sustain their livelihoods), move away from the drug subculture, and stop drug-seeking behaviour.^2,11^ Opioid agonist therapy is generally advocated by individuals who adhere to a harm-reduction approach to drug use. This approach focuses on reducing drug-related risk and harm, rather than on enforcing a prescribed outcome.^14^ Treatment goals in a harm-reduction setting could range from abstinence to reduction or more structured or safer form of use. When dealing with opioid use, treatment goals can be facilitated by opioid agonist therapy.^15^

The approach to managing opioid dependence has moved from focusing on total opioid abstinence towards strategies aimed at harm reduction.^16,17,18^ This may include the use of long-term oral substitution opioid agonists, of which the two most frequently used in South Africa are methadone (full agonist) and buprenorphine (partial agonist).^4,17,19^ The Essential Medicines List (EML) clinical guide is a South African compilation of guidelines and protocols formulated around the WHO’s EML. The purpose of the clinical guide is to assist primary healthcare providers in selecting the most appropriate treatment modalities within the context of health system availability. The EML describes the use of methadone in moderate to severe opioid withdrawal in hospitalised patients. These guidelines follow the more traditional approach to substance withdrawal that aims towards abstinence. They advise that methadone should only be used if clinical symptoms of withdrawal are present, starting at a low dose of 5 mg – 10 mg, with top-up doses as needed.^20,21^ The advised regimen is down-tapering, with the duration of treatment usually lasting 14 days. The withdrawal regimen may be shortened if the patient’s withdrawal symptoms allow it.^8^

Outpatient opioid substitution demonstration and research projects represent the new paradigm in substance use management. These projects do exist in South Africa, but have yet to be adopted in many provinces, as they require infrastructure for daily supervised consumption of substitution opioids and regular follow-up and monitoring due to the risk of accidental overdoses.^18,19^

A concern for medical practitioners with the practice of OST is the fear of overdose, especially if done on an outpatient basis with no control over user behaviour.^4,13^ Highly regulated systems, like the Australian model, have been implemented to facilitate the OST process and decrease risk to users. Doctors are required to undergo mandatory training and registration in order to prescribe OST; individual patient permits are issued, and strict guidelines, including rigid supervised consumption, apply.^2,4,7,11^ Highly regulated systems are associated with low rates of diversion, intravenous use, or deaths due to prescribed medication and provide comprehensive packages of care to patients. However, they are costly and therefore tend to offer only limited treatment spaces. This implies that it would have a limited impact on public health and would be largely impractical for developing countries like South Africa.^2,4,5^

In South Africa, OST is available as part of NGO-funded programmes in Tshwane, Cape Town, Durban, and Johannesburg. However, as services are not all state-subsidised and there are no generic products available to date, the cost of medication remains one of the main barriers to accessing treatment.^14,22^ With the increasing focus on SUDs in public health and the development of multiple outpatient programmes to facilitate the rehabilitation of substance users using OST, the cost and burden to the government will be significantly higher.^23^

The Community Oriented Substance Use Programme (COSUP) provides evidence-based substance use services, focusing on harm reduction and Community Oriented Primary Care (COPC).^15^ Community Oriented Substance Use Programme promotes a paradigm shift to harm reduction through intersectoral collaboration, facilitating accessible comprehensive treatment services at community level, and empowering communities that use substances.^15^ The services provided include screening and assessment for substance use, physical well-being and mental health, brief interventions and referrals, harm reduction counselling, OST, and needle and syringe services (NSP), as well as social services, skills development, food services and shelter. By mid-2019 there were 17 functional and viable COSUP sites available to residents of the City of Tshwane.^15,24^

The strong connection between substance use and homelessness in South Africa has also proven to be a challenge in the substance-use rehabilitation process.^2^ According to a recent estimate by the Human Sciences Resource Council (HSRC), South Africa is home to approximately 200 000 homeless people.^5^ A prospective cohort study done at an emergency department in an urban public hospital in KwaZulu-Natal from November 2016 to September 2017 showed that patients that were homeless had a 40.8% higher rate of unhealthy drug use as compared to the 18.8% housed patients. A total of 16.7% of the homeless interviewed were heroin users and had a lifetime opioid overdose risk of 15.8%.^25^ The Gauteng province has an estimated 25 000 homeless people with approximately 10 000 located in the City of Tshwane.^7^ During the national Level 5 lockdown from March 2020, a total of 241 shelters were made available for the homeless in Gauteng, of which 56 were located in the City of Tshwane.^7^

Treatment services for heroin users are currently fragmented and uncoordinated in South Africa². This is largely due to the poor understanding of the multifaceted nature of drug dependence and the costly treatment process. South Africa should adopt a more integrated and coordinated interdisciplinary approach to substance use, which includes the provision of OST and focussed client participation. The use of methadone for harm reduction in the shelter setting provides a unique perspective on substance use and management that could potentially provide valuable insight into the management and reduction of harm around substance use in the homeless population. This study aimed to describe the doses of methadone used in people receiving OST for the management of acute opioid withdrawal in shelter settings in Tshwane during the national COVID-19 lockdown.

## Research methods and design

This study is a quantitative, cross-sectional, descriptive study. Secondary data obtained during the national COVID-19 lockdown under umbrella ethics approval by the the University of Pretoria (UP) Ethics Committee for emergency research in the homeless shelters. Both existing permanent and new temporary shelters in the City of Tshwane receiving opioid withdrawal management services by the UP Family Medicine Department were included in the study. These were St Wilfrid’s Anglican Church, Capital Park temporary shelter, Lyttleton Sports Park temporary shelter, Reliable House, Melchizedek, Potter’s House, and Koffiehuis shelter.

This study made use of the consecutive daily observed methadone dosing records for the period March 2020 to September 2020. Shelter registers were used to provide information on participants that left the shelter and reasons given on departure. Additionally, COSUP registration records were analysed to access follow-up after leaving the shelters. Records of all substance users (18 years and older) receiving OST for acute opioid withdrawal while staying in shelters in the City of Tshwane during the national lockdown were included in the study. Excluded from the study were all non-substance users, as well as all substance users not receiving OST, while staying in shelters in the City of Tshwane. As part of the harm reduction service, needle-syringe services were also implemented at some of the shelters to reduce the harms associated with injecting drug use.

Demographic data, specifically age, race, and gender were documented from the dosing records, shelter registers, and COSUP registration records. Methadone initiation doses, dosage changes, duration of dose received, and duration of stay in the shelter were also documented. For participants who were weaned, the duration of the weaning process was recorded. Participants that were lost-to-follow-up were documented along with the reasons indicated. Follow-up with COSUP after leaving the shelter was also recorded. When participants left the shelter, they were given a customised COSUP referral letter identifying them as being a recipient of methadone and a previous resident of a shelter. On arrival at a COSUP site, participants presented this letter and, on instruction, the attending medical professional documented them on a separate joining record.

All data collected both digitally via the Phulukisa application, in paper records, and spreadsheets by the family medicine teams that serviced these shelters, were captured in Microsoft Excel spreadsheet that was uploaded to a secure drive. All the parameters were categorical with frequencies and percentages reported by category. Furthermore, cross-tables that enhanced data summary were reported. Some of the objectives were also reached from the latter. Proportions of interest were reported with 95% confidence intervals. Where necessary, testing was done at the 0.05 level of significance.

As this study made use of secondary data, no harm befell participants. During the withdrawal management of substance users living in shelters during the national lockdown, patient autonomy was respected, and patients were informed that the records would be used as data for a future study and if they did not want their information included, it was clearly indicated, and their information was not included in the raw data sheet. All recorded data was made anonymous by only using a numerical identifier in the raw data sheet, protecting the patient’s privacy, and ensuring confidentiality. Additionally, the study did not interfere with day-to-day practices of the shelters, providing continuous harm-reduction and primary healthcare to all clients.

## Results

Daily OST records were received from four permanent and four temporary shelters in the City of Tshwane. A total of 510 patients were documented to have partaken in the harm-reduction programme at the various shelters. Of these, 15 daily patient records were incomplete and excluded from the final analysis, which included 14 male participants, and one female participant.

### Demographic characteristics of the participants

A total sample of 495 participants were included in the final analysis, including 483 men (97.6%) and 12 women (2.4%). The majority of the participants were black (91.9%) and between the ages of 20 and 30 years (71.5%). Table 1 shows the demographic characteristics of participants stratified by gender.

**TABLE 1:** Demographic characteristics of participants stratified by gender.

Variables	Male		Female		All participants	
	*n*	%	*n*	%	*n*	%
**All participants**	483	97.6	12	2.4	495	100.0
**Race**						
Black people	455	91.9	12	2.4	457	92.3
White people	15	3.0	0	0	15	3.0
Mixed race people	20	4.0	0	0	20	4.0
Indian people	3	0.6	0	0	3	0.6
**Age (years)**						
20–30	353	71.5	8	0.02	361	72.9
31–40	97	0.2	4	0.01	101	20.4
41–50	32	6.5	0	0	32	6.5
51 and older	1	0.2	0	0	1	0.2

### Methadone initiation, continuation, and dosage change

**FIGURE 1:**
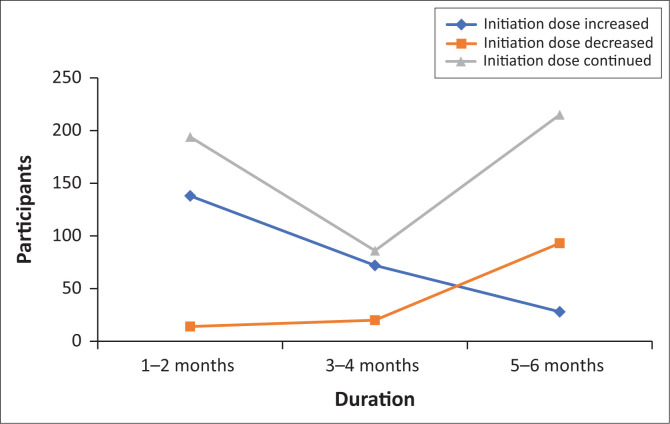
Total change in methadone dose over time.

The initiation doses of participants ranged from as low as 20 mg to the highest dose of 70 mg. Specifically, 64 (12.9%) participants were initiated on 20 mg – 30 mg, followed by 397 (80.2%) initiated on 40 mg – 50 mg, and 34 (6.8%) initiated on 60 mg – 70 mg. A total of 194 (39.2%) participants continued their initiation dose for 1–2 months, after which 138 (27.9%) had their doses increased, and 14 (2.8%) had their doses decreased. Eighty-six (17.4%) participants continued their initiation dose for 3–4 months and 215 (43.4%) continued for 5–6 months. It is noted that 181 (45.6%) participants that continued their initiation dose for 5–6 months were initiated on 40 mg – 50 mg of methadone. Of the 12 female participants, 8 (66.7%) were initiated on 20 mg – 30 mg, 3 (25%) on 40 mg – 50 mg, and 1 (8.3%) on 60 mg – 70 mg of methadone. Three (25%) of the female participants did not stay on their initiation dose for 5–6 months, but it was increased after 1–2 months, of which all were initiated on 20 mg – 30 mg of methadone. Figure 1 demonstrates the total change in methadone dosages over time.

It was noted that, of the 238 (48.1%) participants who had their doses increased, 233 (97.8%) had a total dosage increase of 10 mg – 20 mg. The remaining 5 (2.2%) participants had a total increase of 40 mg – 50 mg. Additionally, on analysis of the overall 127 (25.7%) participants who decreased their doses, it was noted that 97 (77.6%) participants decreased their methadone dose by 10 mg – 20 mg. This was followed by 26 (20.8%) participants who had their doses decreased by 30 mg – 40 mg, and 5 (1.6%) had it decreased by 50 mg – 60 mg.

### Methadone weaning and follow-up

**FIGURE 2:**
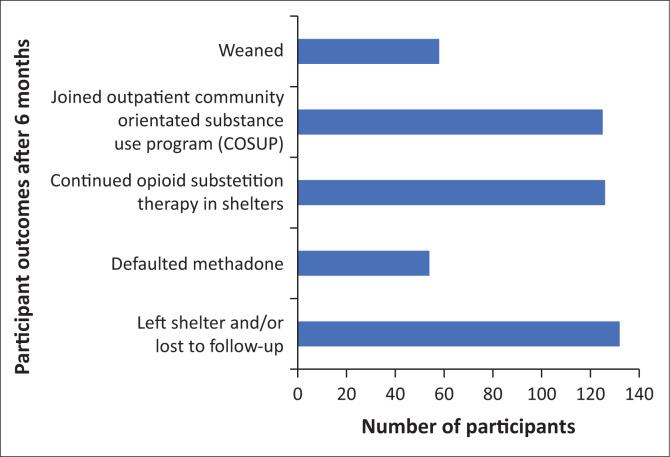
Outcome of participants after 6 months.

Of the 495 participants in the study, 12 (2.4%) were weaned after 1–3 months and 46 (9.3%) after 4–6 months. A total of 437 (88.3%) were not weaned off methadone. However, of these 437, 54 (10.9%) participants stopped collecting methadone abruptly, 100 (20.2%) participants left the shelter prematurely, and 32 (6.5%) participants had no outcome on the daily dotting charts. A total of 126 (25.5%) participants continued to stay in the shelters and received methadone beyond 6 months, with 125 (25.3%) participants leaving the shelter with continued follow-up at a COSUP site. Figure 2 demonstrates the outcomes of the participants after 6 months.

## Discussion

In this study, 397 (80.2%) participants were initiated on 40 mg – 50 mg of methadone, and 34 (6.9%) were initiated on 60 mg – 70 mg. This is significantly higher than the pharmaceutical company recommendations of 10 mg – 30 mg. Equity Pharmaceuticals (the current supplier of methadone in South Africa) recommends a daily initiation dose of 20 mg, equating to 10 mL of a 2 mg/1 mL solution. The shelter initiation doses were also higher than what is recommended in the COSUP standard operating procedure (SOP). The higher initiation doses can potentially be explained by the fact that some participants were initially housed in a larger shelter, Caledonia Stadium, where methadone was used for withdrawal management, prior to being moved to the individual shelters reported on in this study.^26^ Records regarding doses used at Caledonia Stadium were not available for interpretation, but could account for why higher doses were used in individual shelters as these were not true initiation doses, but rather doses people were already on. Another explanation for the higher doses used at initiation could be that the initiators were unfamiliar with the dosing ranges of methadone. Due to the initial chaotic set up and management of the shelters, health professionals from various backgrounds were used to assist with the initiation and distribution of methadone, and the majority were largely unfamiliar with OST practices.

Of the 64 participants that were initiated on the recommended dose of 10 mL – 15 mL, 68.8% had their doses increased after 1–2 months. This was expected as the initiation doses are insufficient to manage withdrawal completely, and recommendations for maintenance doses are between 60 mg and 90 mg as per the methadone package insert.^27^ Although initiation doses of 40 mg – 50 mg are higher than recommended, they are still lower than the recommended maintenance doses. It is, therefore, understandable why this group also had just under 69% of its participants increase their dose after 1–2 months. When lockdown was implemented and people were placed in shelters, many unknown variables were at play. Initially, the idea was to provide short-term withdrawal management and there was no long-term maintenance plan. Most residents only agreed to the methadone because there was no alternative.

Of the 495 participants, 58 (11.7%) were weaned off methadone throughout the 6 months of the study, with the majority of participants, 46 (9.3%), being weaned between 4 and 6 months. Initially weaning was provided on participant request, where a number of participants utilised the shelter setting as a form of inpatient rehabilitation service. This later evolved to where down-tapering and abstinence were promoted and praised, not only among participants, but among providers of OST as well. Despite evidence showing short-term detoxification programmes being less successful, shelters adapted by implementing, what can best be described, as a form of prolonged detoxification. It was noted that most participants that were weaned came from smaller shelters, with multiple participants weaned in the same shelter. The shelters that showed the greatest number of participants weaned had a strong religious influence and focussed on generating a sense of community among those using OST, promoting dose tapering and abstinence. Clients were not forced to wean off methadone, but were given a choice. These participants were weaned in a structured, controlled environment with the absence of external influences, and the majority of them left the shelter without follow-up arrangements. It is unclear whether these participants remained weaned or relapsed in the absence of a controlled environment.

Just over 10% of participants were documented to have stopped OST throughout the 6-month period. This meant that the participant stopped coming for daily dosing of methadone but was reported to still be a resident at the shelter. It is likely that these participants opted to go back to heroin use once lockdown regulations eased. This could also explain why some participants down-tapered their doses.

After the 6-month study period, 125 (25.3%) participants were documented to have joined COSUP and continued with OST. This data were obtained by records generated by the various COSUP sites throughout Tshwane. This result is positive, demonstrating that more than a quarter of participants continued to utilise COSUP services. Providing access to services, improving knowledge about substance use, and familiarising participants with treatment options available are some of the key aspects that contributed to higher continuation of care. Whether the participants that joined COSUP will continue OST long term is beyond the scope of this study.

The participants had access to a multidisciplinary team during their stay in the shelters. One of the most utilised services was social work, allowing participants to re-establish contact with family. Participants leaving the shelters for family reunification was one of the most prevalent reasons given for departure. Other common reasons documented were that participants left voluntarily due to conflicts with other shelter residents, dissatisfaction with ablution and food services, as well as some feeling that they were being mistreated in the shelters. Exploring those allegations was beyond the scope of this study. Thirty-two (6.5%) participants were documented in the final data analysis as having unknown reasons for leaving. The outcome for these participants could not be established in the dosing records reviewed, nor were they captured on COSUP follow-up records. It is suspected that these participants were lost in transition when temporary, smaller shelters were closed, and residents moved to join bigger shelters. After the conclusion of the 6-month study period, 126 (25.5%) participants continued to reside in permanent shelters, receiving daily OST. The continued follow-up and eventual outcome of these participants create an opportunity for further study.

One of the biggest limitations of this study was that the analysis was done on secondary data, thus neither the accuracy of the data, nor the completeness of the data could be guaranteed. Incomplete data were not included in the study, resulting in a smaller study population. Additionally, the study focused specifically on substance use in the homeless. It explored various trends of OST among the homeless population while within a shelter setting, removing many external influencing factors they would normally experience if receiving OST from an outpatient programme. This affected the generalisability of the study, as OST is no longer provided in the same manner to the homeless, as temporary shelters have been disbanded. Comorbid substance use was not assessed or specified in this study, and this may have influenced the methadone doses and high dropout rate. High mental illness comorbidity rate in opioid addicts may have influenced retention and dropout rates; however, comorbid mental illness was not screened for and managed in this study. Additionally, methadone doses were not standardised in the study, but guided by individual clinical assessment which led to the use of much higher doses than recommended by the current National Department of Health’s hospital level Essential Drug List (EDL).

## Conclusion

This study demonstrates high variability in methadone regimes among shelter residents. Initiation doses for most clients were higher than the recommended dose of 10 mg – 30 mg, and may be explained anecdotally by discrepancies in knowledge on OST and incomplete record-keeping of initiation doses. As the lockdown measures eased, many residents chose to leave the shelters, while others remained to receive methadone and other services. The COSUP appears to be effective during periods of increased vulnerability. Despite the seemingly chaotic nature of the shelter setup and methadone initiation, many homeless people who were previously neglected were assisted and have been linked to harm reduction services. The authors acknowledge the need for further study regarding the use of OST in the management of acute opioid withdrawal and recommend proper education and orientation to OST providers to ensure a standardised approach in line with current guidelines.
